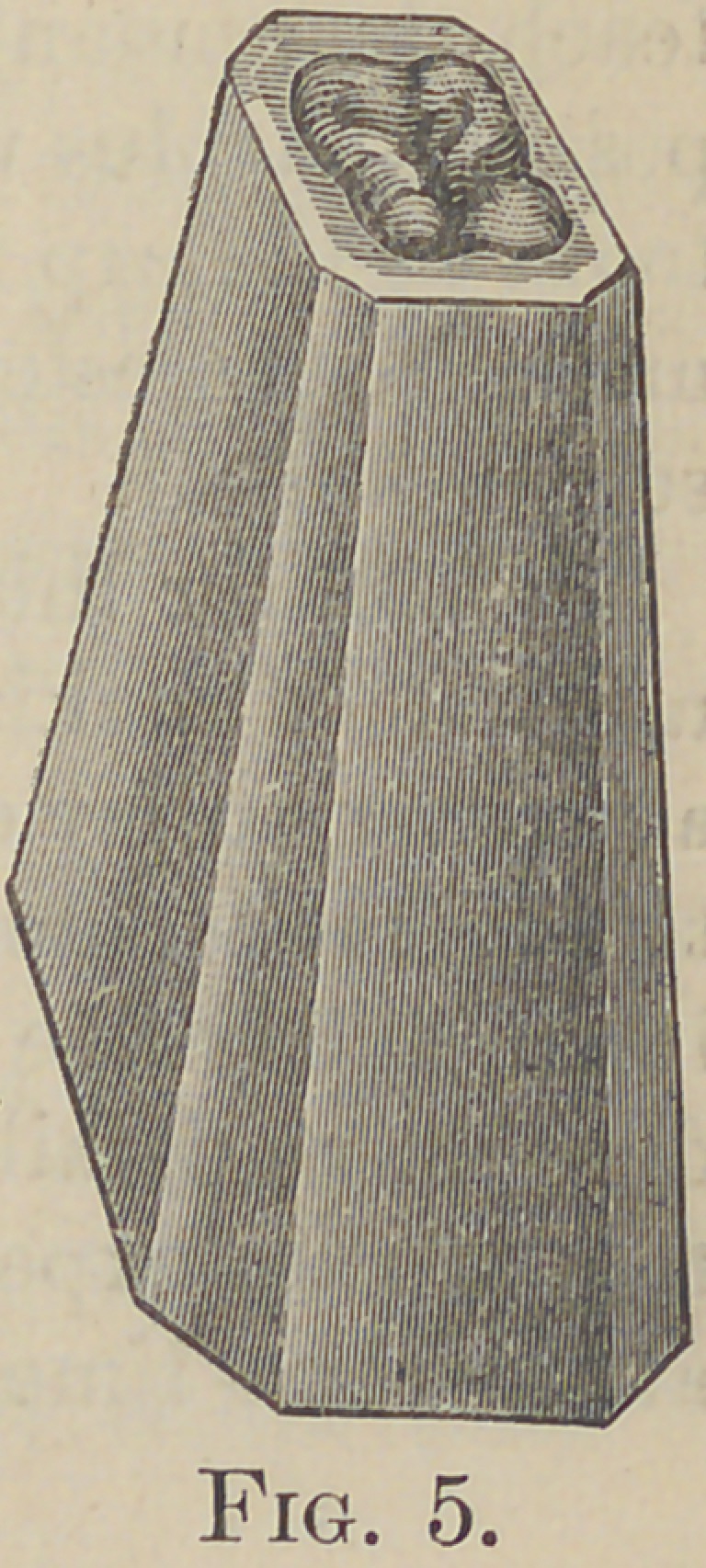# Some Suggestions on Metal Cap Crowns

**Published:** 1888-01

**Authors:** W. Mitchell

**Affiliations:** London, England


### Some Suggestions on Metal Cap Crowns.

BY W. MITCHELL, D.D.S., LONDON, ENGLAND.

[Read before the Ohio State Dental Society, Springfield, Ohio, October, 1887.]

Much has been said, and more has probably been written respecting the various methods of crown work, and possibly as much or more ingenuity has been displayed in this direction than has fallen to the lot of any other portion of our practice.

I do not propose in this short paper to go into the evolution of crown work, as that is already too well known to the educated dentist to need even a passing notice here. Of the various—and I might almost say numerous—methods of crowning teeth or roots, none present to the dentist or patient a greater assurance of success than the metal cap crown, embodying as it does the three essential features requisite to that end, viz., adaptability to the greatest number of cases, cleanliness, and indestructability—always bearing in mind only bicuspids and molars are to be treated by this method.

This process of capping admits of restoring articulating surfaces afforded by no other; for with suitable dies, or only just the addition of a small amount of solder here or there, its capacity in this direction is only limited by that of the dentist. While this subject has been pretty freely discussed both in and out of societies, but little of a practical nature in regard to detail has been touched upon, to enable those of a limited experience to more thoroughly understand the manipulation necessary to the successful completion of this kind of work. It is to that end that this paper is presented.

Taking for granted the tooth or root to be capped is in a healthy condition, *i. e.*, if a devitalized tooth, treatment has been completed and roots filled, I then proceed to prepare the tooth or root for the cap; this is done by various means, principally by corundum wheels of suitable shapes and sizes used on the engine, to reduce in height to allow for restoration of bite or to admit of adjustment between adjacent teeth.

In bicuspids or molars where the root is on a line with the gum I find it a good plan to put one, two, and sometimes three good platina pins in the pulp canals, fitting them first, make them the proper length and then drive them solidly home, they thus afford much support to the cap, especially where it has a good deal of work to perform.

After having properly coned the tooth or root, I then go all round the cervical margin with an instrument represented in Fig.

FIG. 1.

1, removing every vestige of enamel remaining there, this is easily accomplished by inserting the point of the instrument first under the free margin of the gum, and with a draw cut, the enamel is trimmed off very rapidly and effectually. It will be observed that convexity of the distal surface of the instruments prevents injury to the gum margin.

The thorough preparation of the neck of the tooth is one of the most important factors in the success of the operation, for upon this, the fit and retention of the cap almost entirely depends; hence the absolute necessity for its most careful observance. This done, the tooth is ready for the cap. For this I use coin gold rolled to No. 5 B. P. G. for the band, and No. 3 for the top. The reason I prefer coin gold, it works more uniformly than anything else I have tried, and its color is good in the mouth, besides being quite hard enough to withstand all service it may be subjected to.

In preparing the band I find no model—except the tooth itself—necessary, all others being "a delusion and a snare." I then take a piece of ordinary visiting card and approximate its length and height, then cut plate as required—a piece of heavy lead foil is a good thing for a pattern also—then with a pair of medium sized pliers with one jaw flat and the other half round, Fig. 2, I proceed to adapt the plate to the tooth making the band slightly flaring towards the grinding surface. When I have fitted the band as well as possible, close it a little by squeezing between the fingers so it springs over the tooth when put on for trial, then should it not pass evenly under the gum, trim the band till it follows the contour of the gum; I find a pair of nail scissors with short but heavy blades the best for cutting the gold used in this work; then having left a line or so for lapping, always having the lap come on the outside for convenience in marking and trimming, mark with the point of Fig. 1 along the lap, remove the band from the tooth, close it about a line, then solder, this is most easily accomplished over an ordinary bench bunsen, holding the work with a pair of solder pliers, flush the lap nicely; I prefer a lap joint to an edge to edge one, as it is easier to make, can be made just as neatly, and proves a great saving in both time and patience.

FIG. 2.

Having soldered the band try it on, if it is too tight, place it on the horn of an anvil, when a few light blows with a hammer along the lap will soon stretch it to the required size, and owing to the softness of the gold, in a great many instances it will be found the band may be driven on nicely without any stretching.

Should the lap on the inside need any attention after soldering, a half round file judiciously applied will soon set that all right. I then champfer the edge of the band that goes under the gum, and burnish it, as it then more readily insinuates itself between the gum and the tooth. After the band is started I assist it to where I ultimately want it by driving it on with a steel mallet, using a piece of apple wood or bone about five inches long, checkered on the end to prevent slipping, to assist it to its place.

I then judge as to the height the band is to be left, marking it, if necessary to be trimmed, remove, and trim, if necessary, then go around the band with the contour pliers, Fig. 3, these produce the result, Fig. 4; these pliers I designed after trying other methods of contouring, and not being satisfied with the results, these pliers I have found to meet all the requirements of the case.

FIG. 3.

After contouring I take the band and hold its top edge against the flat side of a corundum wheel on the lathe; and by giving the band a slight rotary motion as it is held between the thumb and fingers, a flat surface is secured, it is then ready for the top.

FIG. 4.

The simplest method I have found for producing these is to take teeth that have been extracted, or models from impressions of natural teeth, investing the roots and crowns in plaster, almost to the grinding surface, leaving the block about two inches high, this is trimmed leaving a base about an inch square. I find squaring, then champfering the corners the best shape for drawing, the face of the block is made flat and left one-sixteenth of an inch wide, this is to act as a guide in the finished die, to better assist the operator in utilizing his gold, and later on as a means of holding the top while soldering. After securing the model as described, a sand—or what is better, marble dust—mould is made, this is poured with zinc, which is finished with file and graver, then polished on a lathe.

For the benefit of those who, from some cerebral defect have allowed themselves to believe dental mechanics *infra dig*, would say, all this part of the process can be done by any smart assistant quite as well as by the possessor of the giant intellect, who fain would shun the essential detail whereby he is placed in a position to receive the fee that others probably have done most to place within his reach. Models for crowns can so easily be made by the process described that any case may easily be contended with. I find by having a typical set of crowns, and a number of irregular shapes, or by simply turning a die around, or only partly so, while swaging almost any combination of articulating surface may be produced.

Another reason why this method is a good one, is its simplicity and cheapness, any one capable of making a die can succeed in any case, something that cannot be said of many of the elaborate, costly, and not always time-saving appliances that are on the market. The die having been made as described will appear as in Fig. 5.

FIG. 5.

No counter die is needed for producing the tops, simply place the gold to be stamped on a piece of lead, a smooth surface can easily be obtained by a blow from a hammer. I find it best in striking up the top to put a piece of heavy tin foil between the gold and lead, this prevents the possibility of any small particle of lead adhering to the gold and producing sweating during the subsequent stages of soldering. After swaging the top the flat margin can be utilized by holding it during soldering, (as mentioned in previons stage of the process,) flush the top with 20 karat solder, this can be done, as with the band, over a bench bunsen burner. Having done this, touch the flat edge of the band with borax, placed in the desired position on the top, return to the flame, when top and band will soon unite in the most satisfactory manner, drop into pickle while hot to remove borax, trim off surplus plate with shears, then grind off overhanging edges to the required contour, polish same as a gold plate—minus stoning down—and the cap is ready for final adaptation.

It will probably be remarked that no provision has been made for the exit of surplus cement; I think that experience will teach that no special vent is needed, for no matter how perfectly one may appear to have the cap fitted, the cement will find its way out if mixed to the proper consistency.

The best cements I have found to use in this work are Richmond's Crown Cement, and Welch's Crown Cement, both kinds producing good results, owing to their smoothness in mixing, and a tendency not to set too rapidly. The cement should be mixed so it will just drop from the spatula, a little experience will soon teach the amount requisite to fill the cap and leave the least possible surplus which will find its way out round the neck of the tooth as the cap is driven home, this is best accomplished by the method suggested for setting the band, the rest of the operation suggests itself.

I claim for this method it is at once the most simple and easy method of making metal cap crown known, never necessitating a greater outlay of time than an hour and a half to prepare a tooth, make, and adjust crown, and I have made one and fitted it in thirty-five minutes, thus demonstrating that although descriptive detail involves some little time, the practical application of it in experienced hands reduces time to the minimum, and at the same time secures the most satisfactory results.

## Figures and Tables

**Fig. 1. f1:**
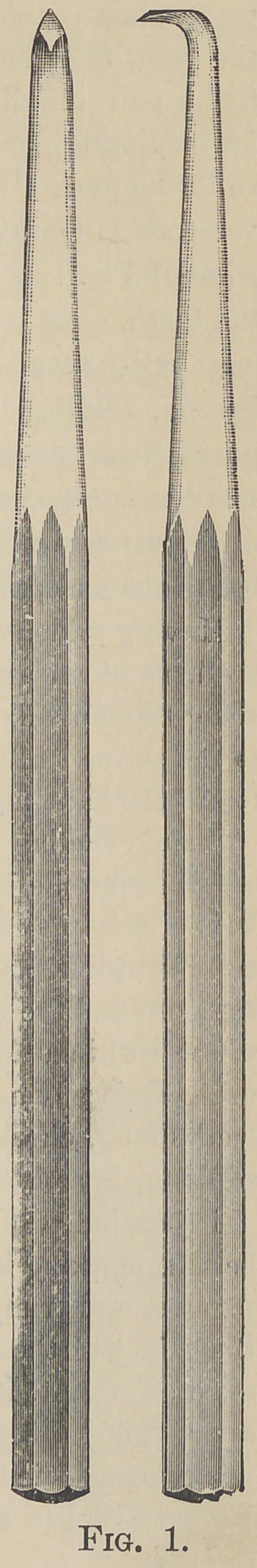


**Fig. 2. f2:**
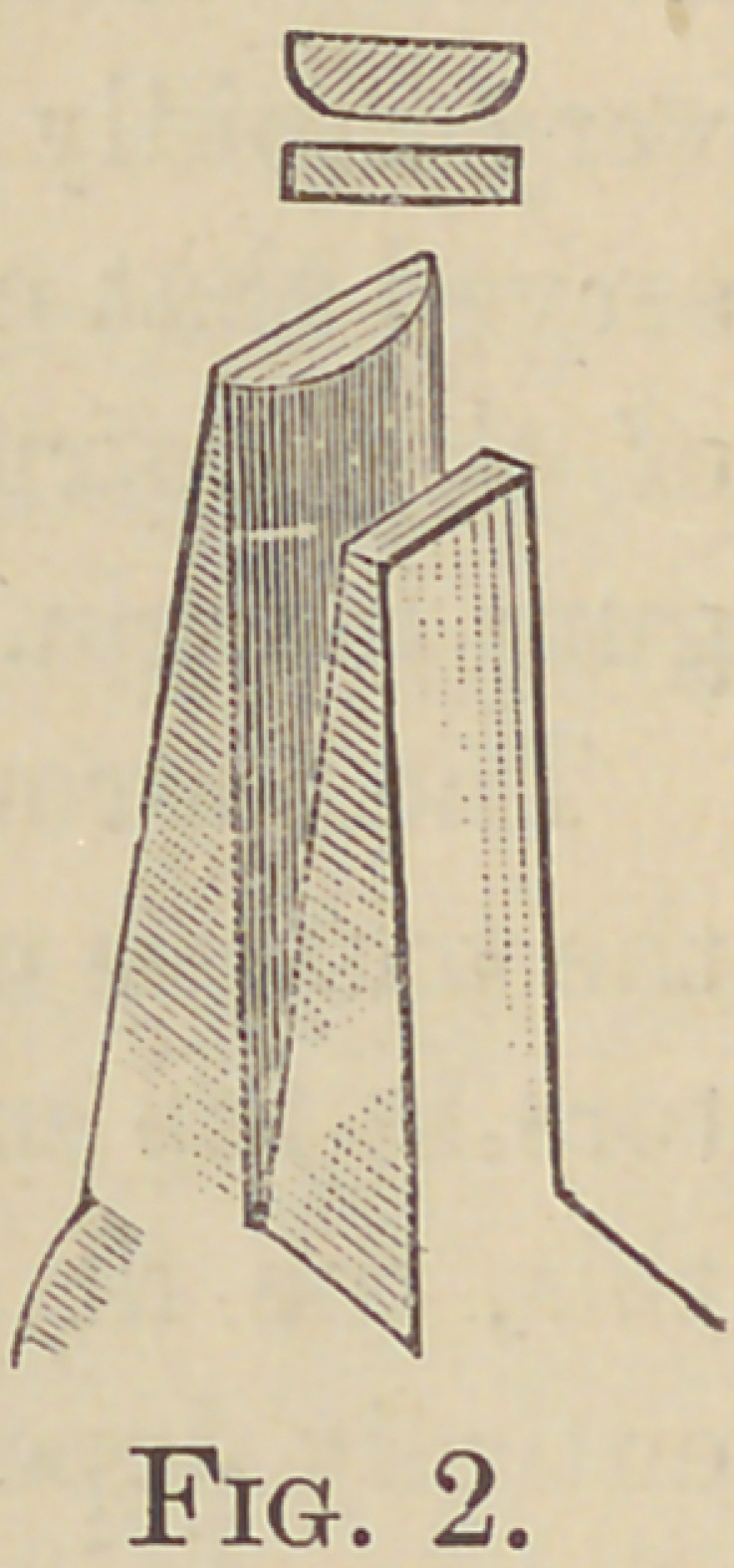


**Fig. 3. f3:**
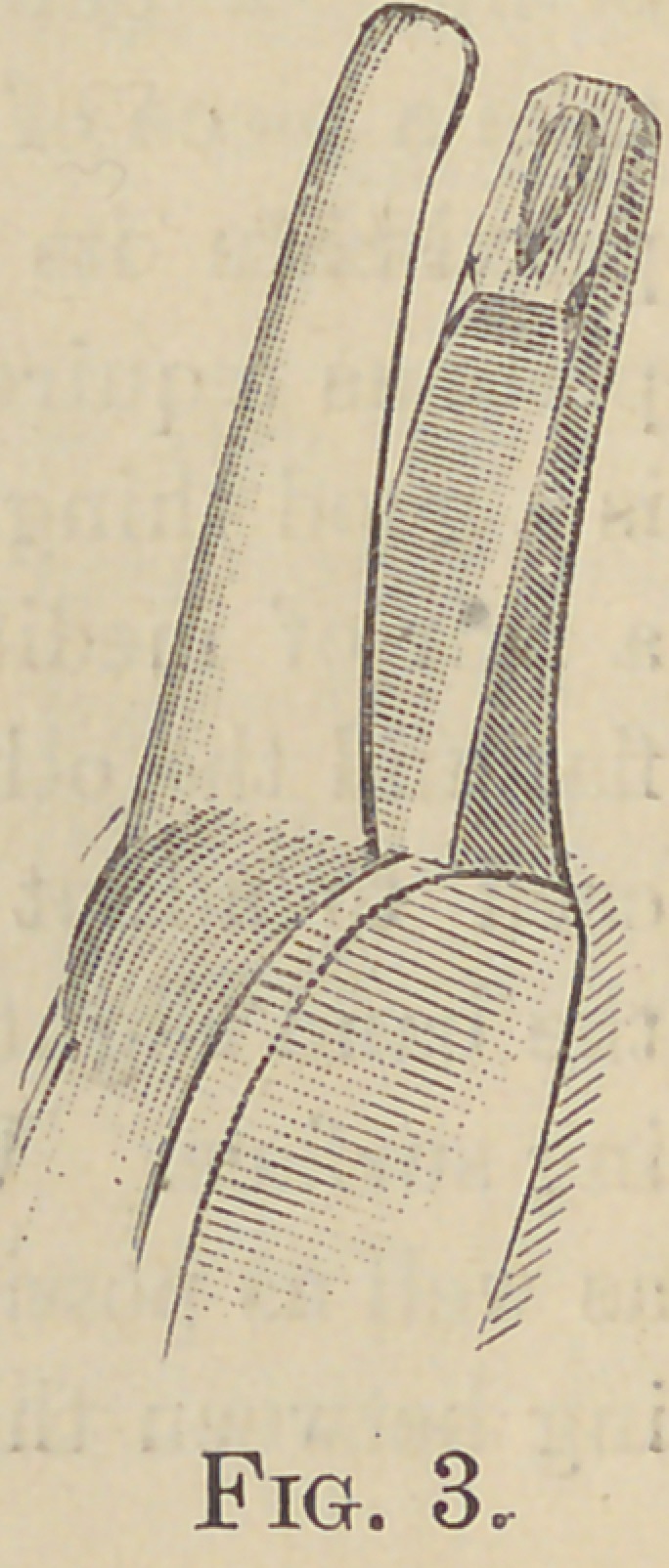


**Fig. 4. f4:**
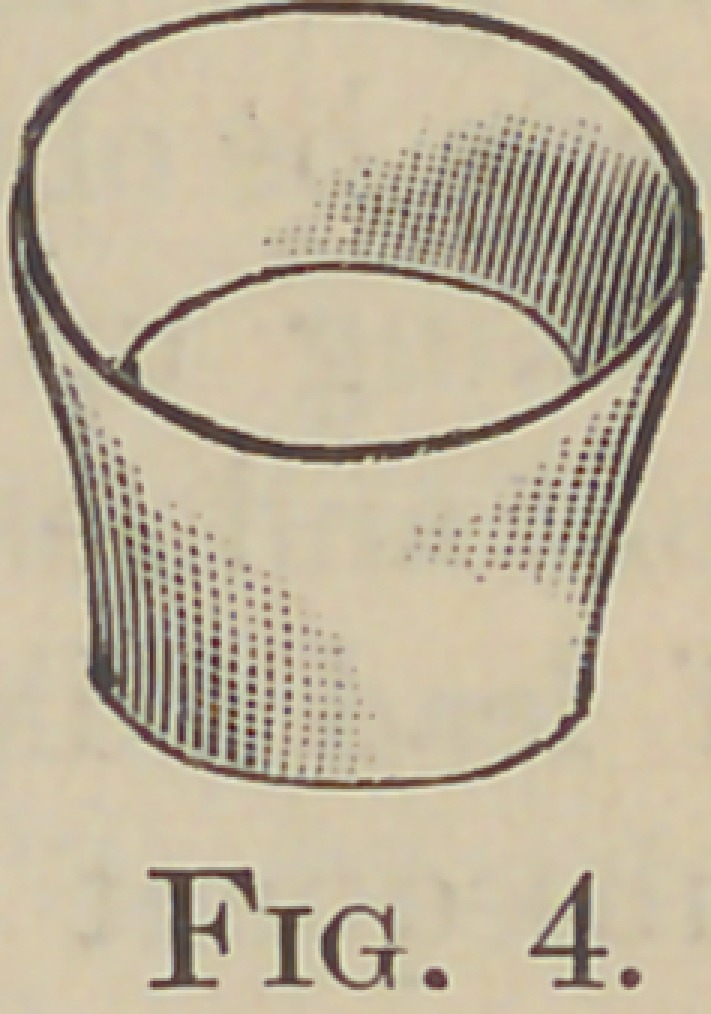


**Fig. 5. f5:**